# Correction: Green synthesis of 1,4-benzodiazepines over La_2_O_3_ and La(OH)_3_ catalysts: possibility of Langmuir–Hinshelwood adsorption

**DOI:** 10.1039/c8ra90005a

**Published:** 2018-01-29

**Authors:** Archana Singh, Veerabhadraiah Palakollu, Aman Pandey, Sriram Kanvah, Sudhanshu Sharma

**Affiliations:** Department of Chemistry, Indian Institute of Technology Gandhinagar Palaj Gandhinagar Gujarat-382355 India ssharma@iitgn.ac.in kanvah@gatech.edu +91-79-2397-2324 +91-79-2397-2583 +91-7819859706

## Abstract

Correction for ‘Green synthesis of 1,4-benzodiazepines over La_2_O_3_ and La(OH)_3_ catalysts: possibility of Langmuir–Hinshelwood adsorption’ by Archana Singh *et al.*, *RSC Adv.*, 2016, **6**, 103455–103462.

The authors regret that in the original manuscript there were some errors in the structures displayed in Scheme 1 and Table 1. The correct scheme and table are presented herein.

**Scheme 1:**
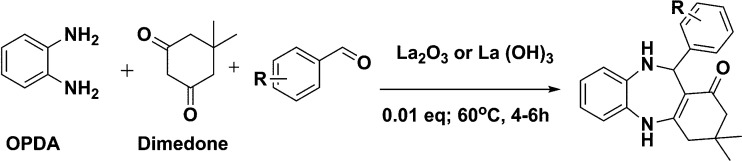
Schematic of a synthetic strategy for preparation of 1,4-benzodiazepine derivatives.

**Table 1:** List of the reactions performed with the different aldehydes, their reaction times and the isolated product yield

Entry	Reactant A	Reactant B	Reactant C	Product	Time (h) La_2_O_3_/La(OH)_3_	Yield (%) La_2_O_3_/La(OH)_3_
1	OPDA	Dimedone	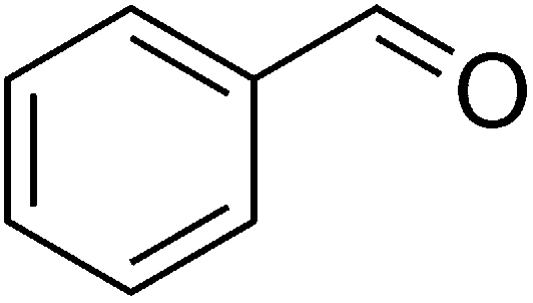	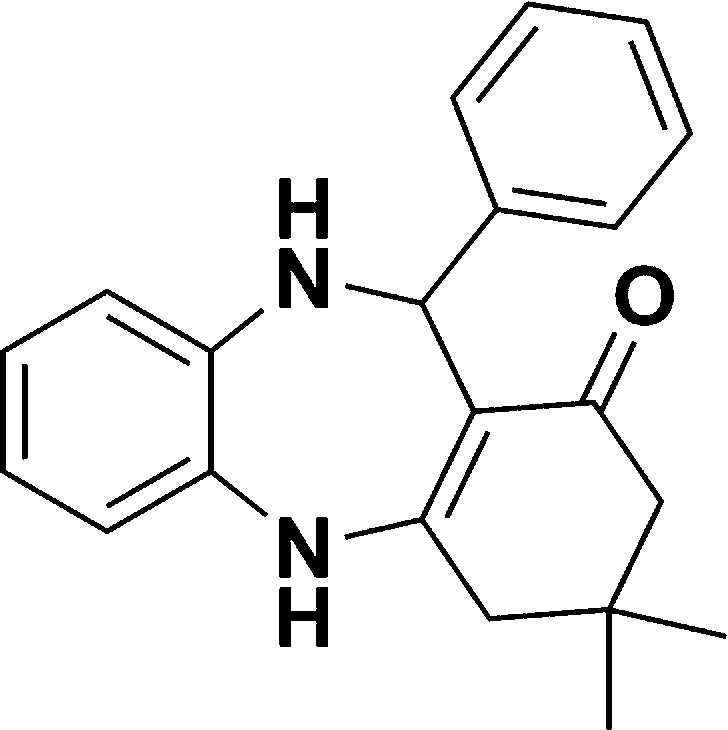	4/3.5	81/76
2	OPDA	Dimedone	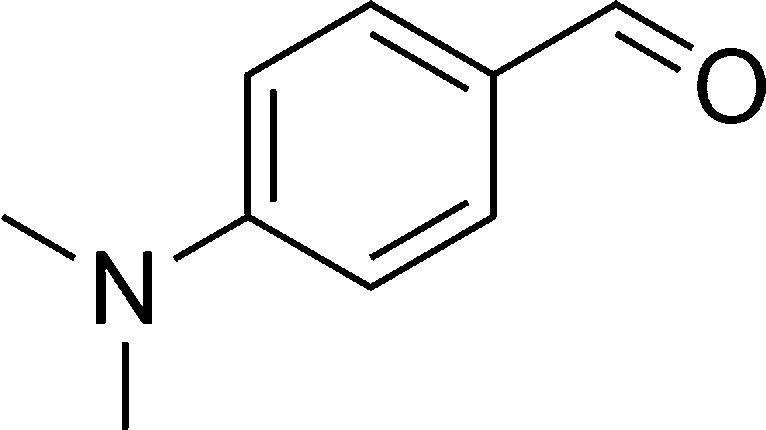	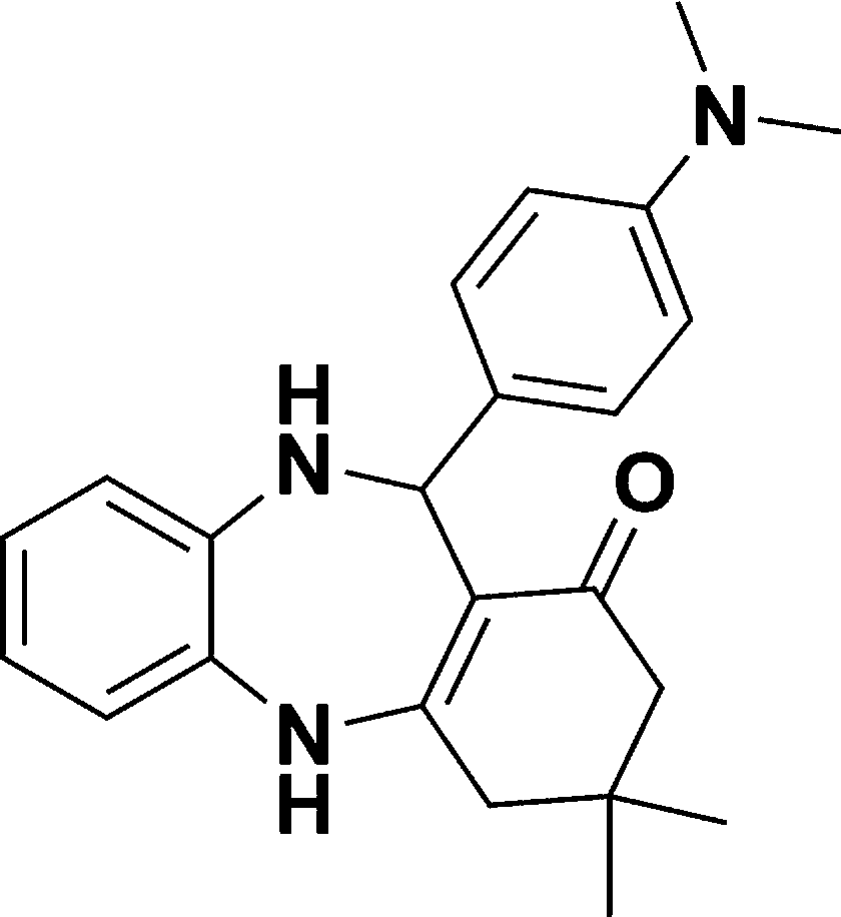	4.6/5	79/82
3	OPDA	Dimedone	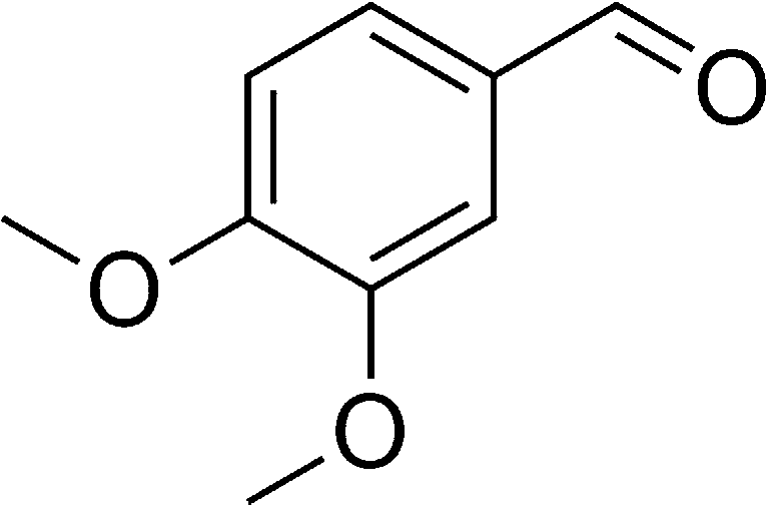	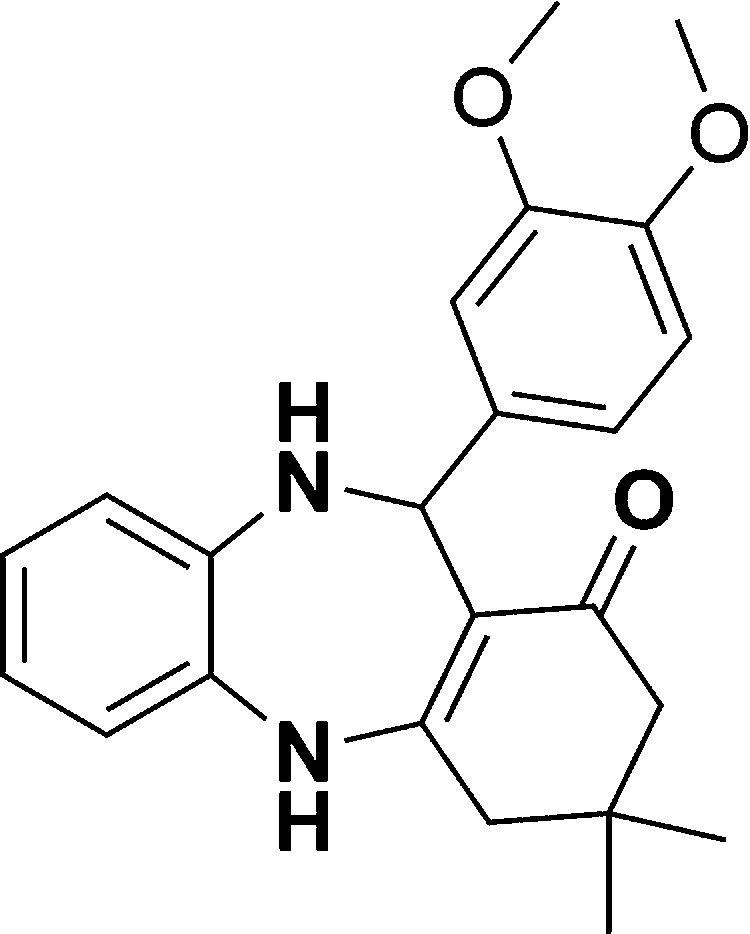	4.5/4.5	80/76
4	OPDA	Dimedone	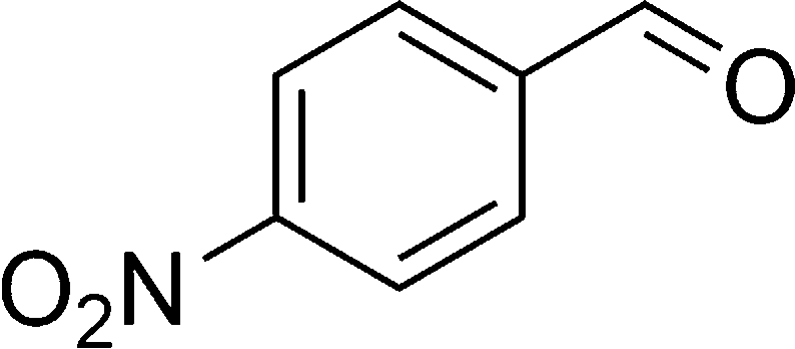	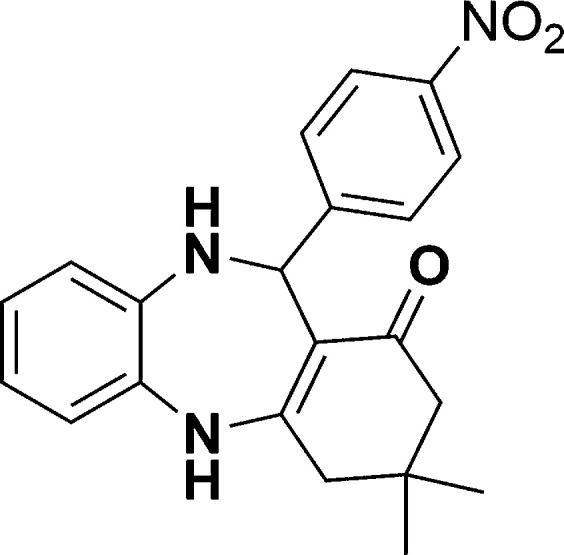	3.5/3	83/85
5	OPDA	Dimedone	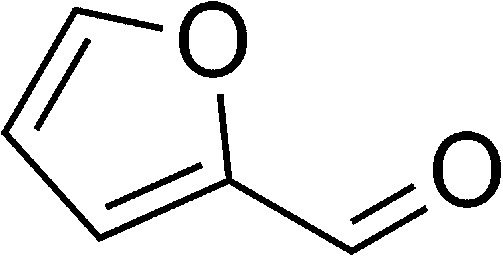	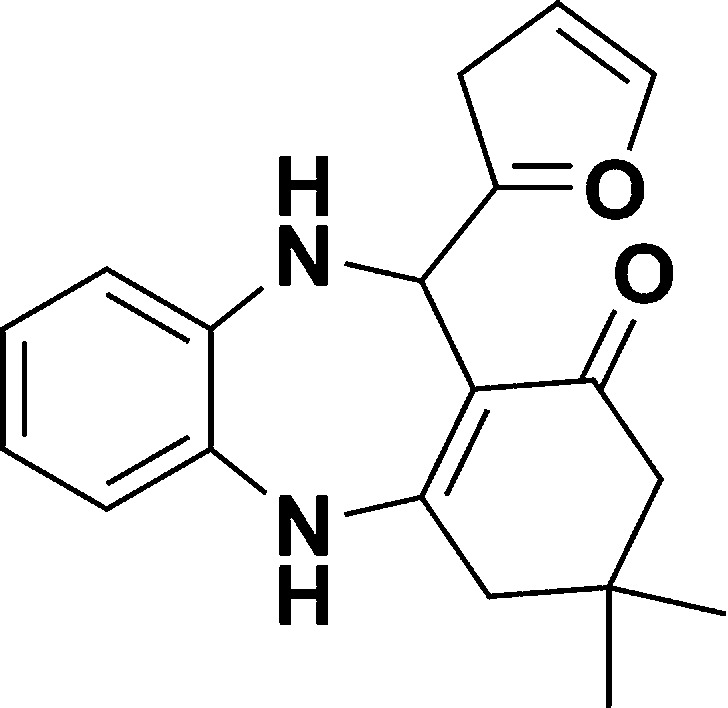	4.5/5	81/79
6	OPDA	Dimedone	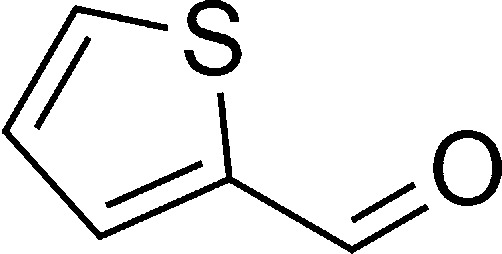	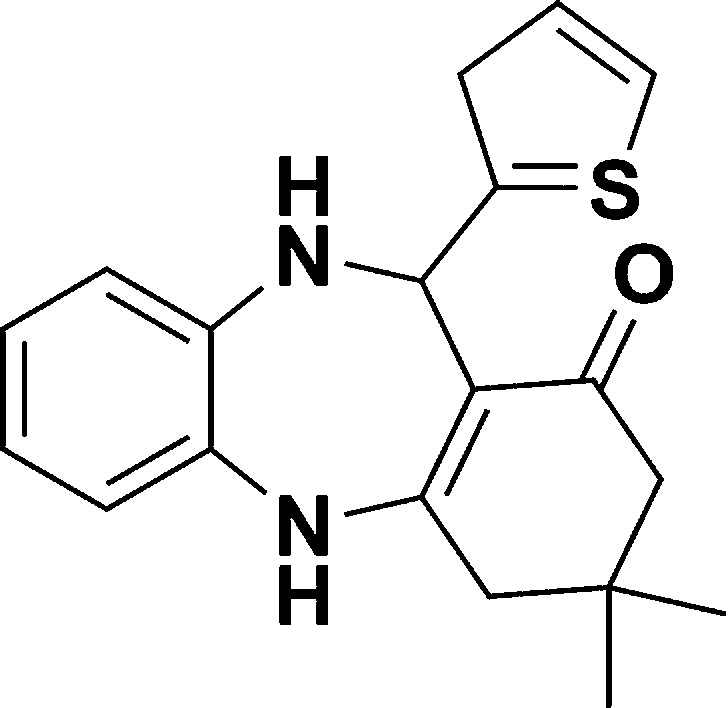	4.8/5	76/79
7	OPDA	Dimedone	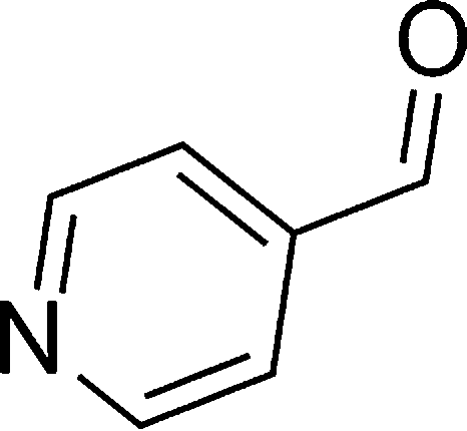	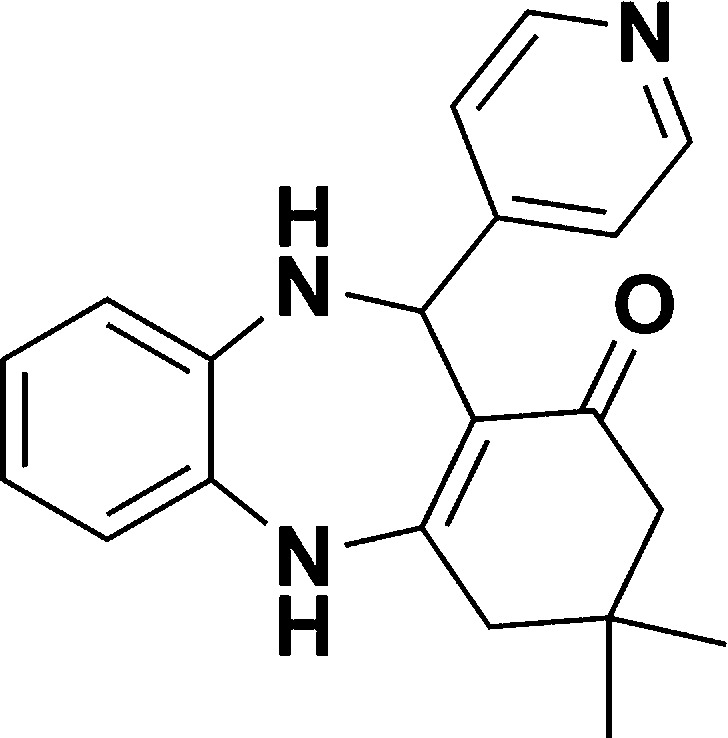	5/4.5	74/76
8	OPDA	Dimedone	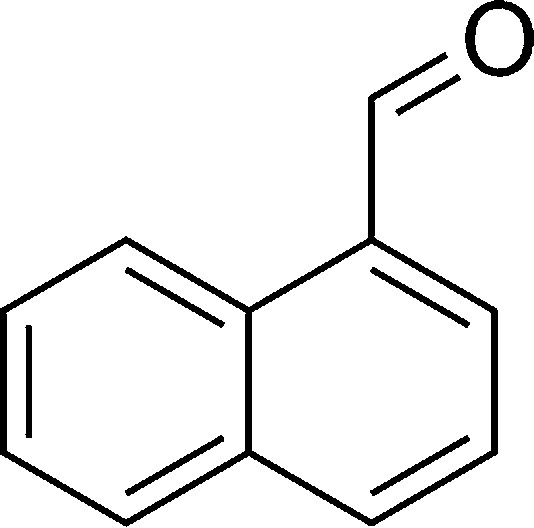	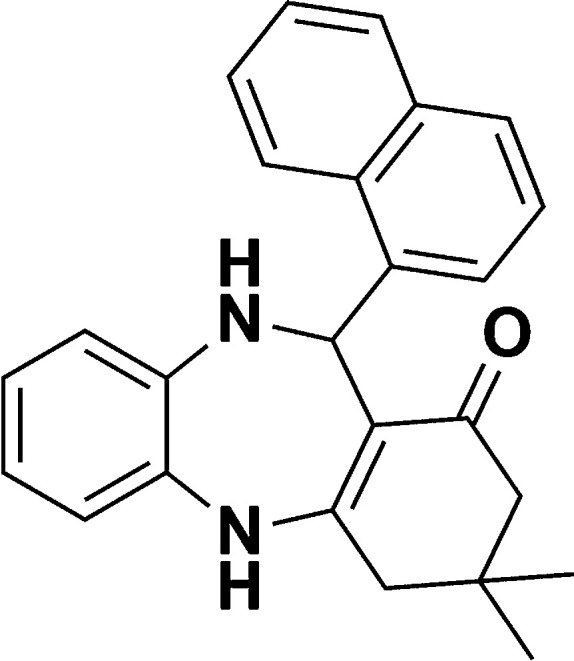	4/3.5	69/71
9	OPDA	Dimedone	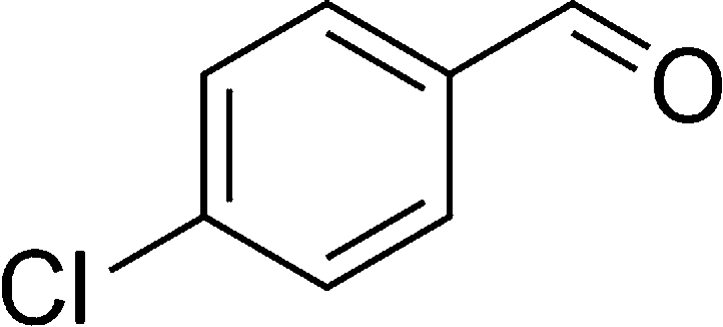	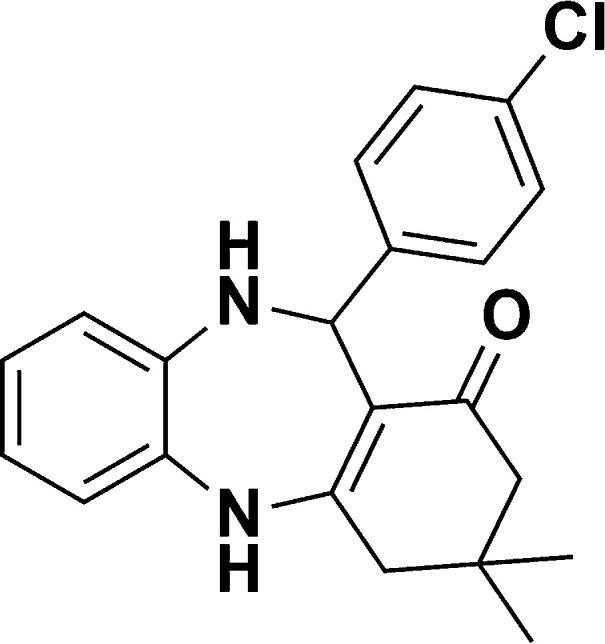	3.6/4	84/81
10	OPDA	Dimedone	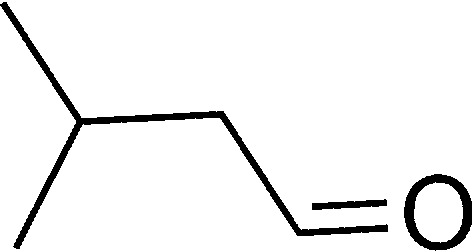	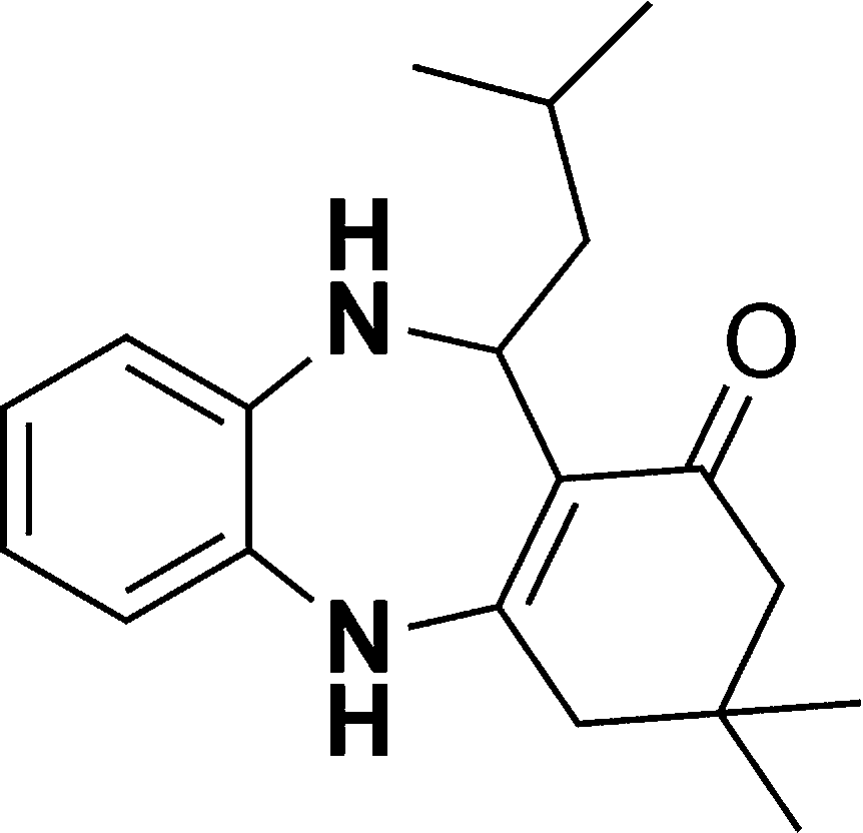	5/4.5	75/70

The Royal Society of Chemistry apologises for these errors and any consequent inconvenience to authors and readers.

## Supplementary Material

